# Sprinting After Having Sprinted: Prior High-Intensity Stochastic Cycling Impairs the Winning Strike for Gold

**DOI:** 10.3389/fphys.2019.00100

**Published:** 2019-02-14

**Authors:** Naroa Etxebarria, Steve A. Ingham, Richard A. Ferguson, David J. Bentley, David B. Pyne

**Affiliations:** ^1^Research Institute of Sport and Exercise, Faculty of Health, University of Canberra, Canberra, ACT, Australia; ^2^Supporting Champions, London, United Kingdom; ^3^School of Sport, Exercise and Health Sciences, Loughborough University, Loughborough, United Kingdom; ^4^College of Nursing and Health Sciences, Flinders University, Adelaide, SA, Australia; ^5^Australian Institute of Sport, Canberra, ACT, Australia

**Keywords:** repeated sprints, stochastic cycling, peak power, race profile, triathlon

## Abstract

Bunch riding in closed circuit cycling courses and some track cycling events are often typified by highly variable power output and a maximal sprint to the finish. How criterium style race demands affect final sprint performance however, is unclear. We studied the effects of 1 h variable power cycling on a subsequent maximal 30 s sprint in the laboratory. Nine well-trained male cyclists/triathletes (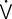
O_2peak_ 4.9 ± 0.4 L⋅min^-1^; mean ± SD) performed two 1 h cycling trials in a randomized order with either a constant (CON) or variable (VAR) power output matched for mean power output. The VAR protocol comprised intervals of varying intensities (40–135% of maximal aerobic power) and durations (10 to 90 s). A 30 s maximal sprint was performed before and immediately after each 1 h cycling trial. When compared with CON, there was a greater reduction in peak (-5.1 ± 6.1%; mean ± 90% confidence limits) and mean (-5.9 ± 5.2%) power output during the 30 s sprint after the 1 h VAR cycle. Variable power cycling, commonly encountered during criterium and triathlon races can impair an optimal final sprint, potentially compromising race performance. Athletes, coaches, and staff should evaluate training (to improve repeat sprint-ability) and race-day strategies (minimize power variability) to optimize the final sprint.

## Introduction

Modern road cycling events held in large cities commonly use closed circuit criterium style cycle courses during major competitions such as a World Cup, World Championship, or Olympic Games. Consequently these race-courses often consist of repeat laps of numerous tight and technical corners that result in multiple rapid accelerations and decelerations (Menaspa et al., 2017), often close to and above maximal aerobic power (Etxebarria et al., 2014b). The combination of geographical, technical and tactical characteristics of these cycling races can elicit a variable power profile (Ebert et al., 2006; Bernard et al., 2009). Other Olympic events resembling multiple high intensity efforts before a final sprint include the scratch and also the points race in track cycling or even the ∼1 h cycle section of modern draft-legal Olympic distance triathlon races. However, it is unclear how the modern race settings affect the physiological and performance abilities of the athletes compared to the traditional ‘out and back’ style road cycling courses. Furthermore, it is worth exploring how well athletes are prepared physically to suit modern race demands, and also track cycling events such as the scratch and points race.

Despite the relatively extensive analysis of multiple-stage road cycling races, little is known about the shorter road cycle races such as time trial events, with no previous study to our knowledge having investigated the effects of highly variable power output cycling on the ability to generate a short-term maximal sprint. Criterium style courses characterized by a mass start, frequent tight corners and bunch riding, can result in highly variable intensity cycling exercise that increase the physiological demands compared with less variable non-drafting or time trial events (Etxebarria et al., 2013). Moreover, the > 40 min race performance of closed circuit technical courses are best associated with the cyclist’s ability to generate high power output over efforts less than 2 min long, showcasing the importance of short-term power output for these endurance events (Babault et al., 2018). These type of races often contain breakaways that demand a sustained high intensity burst, often preceded by multiple high intensity efforts (Abbiss et al., 2013), and followed by a final decisive sprint (Peiffer et al., 2018). Similarly, many track cycling events have a pattern of multiple high intensity efforts throughout, and are often contested in a sprint to the finish line. Multiple sprints without adequate recovery in between lead to lowered repeat sprint ability (Gaitanos et al., 1993), and this could be detrimental to a rider’s final sprint where the race is typically decided.

Cycling power profiles can be readily assessed with use of a power-meter in training and during competition, however, simplified summaries of power analysis do not often reflect the demands of the session (Passfield et al., 2017). The highly variable power output of criterium-style races include multiple 10–30 s high intensity efforts and ∼12% of the time spent at exercise intensities above 8 W⋅kg^-1^ (Ebert et al., 2006), during which the physiological demands differ markedly from time trial style cycling (Etxebarria et al., 2014c). Furthermore, draft legal Olympic distance triathlon races can induce high intensity power outputs for a substantial time (∼15%) including frequent high intensity efforts spread intermittently throughout (Bernard et al., 2009). These reoccurring high intensity efforts often exceed supra-maximal intensities ranging 100 to 140% of maximal aerobic power (Etxebarria et al., 2014b). With frequent high intensity efforts, the physiological demands of city-based certain road cycling events and draft legal triathlon are similar (Ebert et al., 2006).

The physiological demands imposed by different cycling strategies including constant and variable power cycling have been studied in a laboratory setting by implementing variable power protocols with smaller variability (Palmer et al., 1997; Lepers et al., 2008; Suriano et al., 2010) than that observed in some contemporary cycling races (Ebert et al., 2006; Bernard et al., 2009). The duration of the treatment protocols implemented (variable vs. constant) also differ substantially between studies: from ∼30 min (Bernard et al., 2007; Suriano et al., 2007; Lepers et al., 2008; Thomas et al., 2012) to ∼2 h (Palmer et al., 1999) and use a diverse range of performance and outcome measures. These methodological differences between studies investigating constant and variable cycling limit the transfer of the findings to actual sporting performances. A recent laboratory-based protocol with a range of power variations showed substantially greater physiological demands during variable power cycling compared to a sustained effort matched for mean power output (Etxebarria et al., 2013). However, no cycling performance outcome measures were reported and it is unclear how sprint ability would be affected by multiple intermittent high intensity efforts.

Many of these road (and selected track) cycling races are decided in a bunch-sprint after a multiple of high intensity efforts, raising the need to conserve repeat sprint-ability to minimize fatigue before the final sprint that can last ∼20 s or more (Peiffer et al., 2018). As a secondary performance measure for Olympic distance triathlon, the impairment of a maximal cycling sprint could translate to accumulated fatigue before the subsequent and decisive 10 km run. Therefore, the aim of this study was to compare the effect of 1 h cycling at variable power (simulating real world competition demands) vs. constant power output, matched for time and mean power output, on the ability to generate maximal power during a subsequent 30 s sprint. This information will inform the preparation for, and tactics employed during, different cycling and triathlon events.

## Materials and Methods

### Subjects

Nine well-trained male triathletes and cyclists (age: 30 ± 7 year; stature: 1.79 ± 0.05 m; body mass: 74.3 ± 5.3 kg; 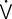
O_2peak_ 4.9 ± 0.4 L⋅min^-1^/66.0 ± 3.9 mL⋅kg^-1^ min^-1^, mean ± SD) completed the preliminary testing and experimental cycle trials in a single group cross-over design. All subjects had at least 3 years of training and racing in cycling and triathlon events. In the 24 h prior to each laboratory visit subjects were required to abstain from any physical exercise, caffeine and alcohol intake and replicate the same dietary practice. The study was approved by the Loughborough University Ethics Advisory Committee and followed the guidelines of the Declaration of Helsinki. All subjects provided written informed consent after explanation of the study protocols and experimental procedures.

### Procedures

All participants reported to the laboratory on three separate occasions. The first visit involved performing an incremental exercise test to determine 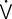
O_2peak_ followed by a 30 min break and a 30 s maximal sprint familiarization trial. The subsequent two visits comprized of a 1 h cycle at either variable (VAR) or constant power (CON) cycling, in a randomized counterbalanced order, with a 30 s maximal sprint just before and closely after the 1 h cycle. The two 1 h experimental cycle trials were performed on two subsequent occasions and at least 5 days apart. The preliminary 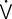
O_2peak_ test consisted of a progressive incremental ramp test on an SRM cycle ergometer (SRM Ergometer with integrated SRM Training System, Science version, Jülich, Germany) following a 10 min warm up at 100 W. The starting power output for the maximal test was between 160 and 180 W, depending on the level of training and experience of subjects. Increments of 5 W every 15 s were employed during the maximal progressive test to ensure exhaustion was reached after approximately 10 min. This protocol allows for a higher maximal aerobic power for a similar 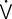
O_2peak_ than a more traditional 3 min incremental stage protocol (Bishop et al., 1998). Pedal cadence was freely chosen and maintained at a constant rate. Maximal aerobic power was defined as the mean of the highest consecutive power values recorded during the test for a 1 min period. Participants performed a 30 s maximal sprint familiarization trial, 30 min after the 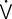
O_2peak_ test.

**FIGURE 1:**
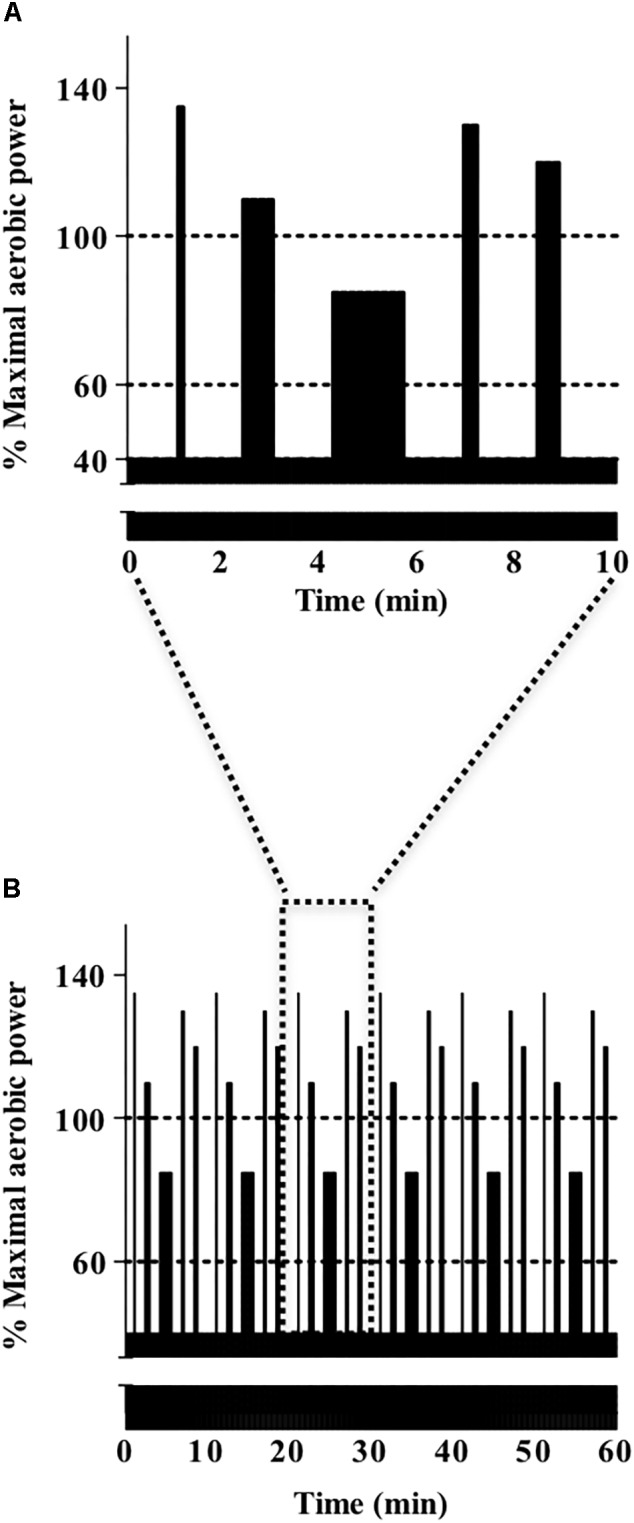
**(A)** A representative 10 min section of the 1 h variable power protocol showing five short higher intensity intervals. **(B)** The 1 h power protocol for the variable power experimental trial including 30 efforts ranging between 10 and 90 s and exercise intensities between 40 and 135% maximal aerobic power.

Upon arrival for the CON or VAR trial subjects performed a 10 min warm up at 100 W followed by a 30 s maximal sprint from a stationary start before the 1 h cycle trials. Due to the lack of information in criterium style races, the mean intensity for both cycle trials was set at 60% maximal aerobic power, similar to the intensity observed during draft legal triathlon races (Le Meur et al., 2009). The CON trial involved cycling for 1 h at a constant power equivalent to 60% maximal aerobic power. The power variations during VAR were characterized by intermittent efforts of different intensities and durations: 10 s at 135%, 40 s at 110%, 90 s at 85%, 20 s at 130%, and 30 s at 120% of maximal aerobic power (Figure 1). This protocol was designed to replicate a generic power profile typically experienced during criterium races and the cycle section of triathlon races, and similar to the Beijing Olympic test event (Bernard et al., 2009). Subjects self-selected their preferred pedal cadence during the first trial and were required to replicate this during the second trial. A minute after terminating the 1 h cycle test subjects performed another 30 s maximal sprint. Participants were allowed to drink a maximum of 750 mL of water during the 1 h cycling trials.

Body mass was recorded on the first visit to the laboratory using an electronic scale (Seca 770, GMBH & Co., Germany). Peak heart rate (HR_peak_) was recorded during the 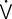
O_2peak_ incremental exercise test using a telemetry system (Polar Electro iS610, Oulu, Finland). A 25 μL capillary blood sample taken from a finger-tip within 30 s of the end of the maximal test and analyzed for blood lactate concentration (B_La_) using an automated blood lactate analyzer (Biosen C-Line, Southam, United Kingdom). During the 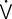
O_2peak_ test expired air was sampled continuously for CO_2_ and O_2_ content and volume on a gas analysis system with a day-to-day reliability of 2.3% (Oxycon Pro Jaeger, Höchberg, Germany). Respiratory gas exchange variables (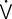
O_2peak_, 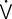
CO_2_, 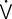
_E_ ) were sampled every 15 s, the mean of the highest consecutive four readings taken as the 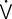
O_2peak_ value.

Power output during the 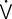
O_2peak_ incremental exercise test, 30 s maximal sprints and 1 h cycling trials were measured on an electromagnetically-braked cycle ergometer, hyperbolic mode. During the sprint test, the ergometer was set in open test mode. The cycle-ergometer set up was individualized, mimicking each participant’s bike set up on their own bike. Cycling data were downloaded to a computer and analyzed with SRM software (v6.40.05, Schoberer Rad Meßtechnik, Germany). The SRM power-meter was zeroed before each trial by recording the zero offset without any force/load on the cranks. During the 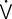
O_2peak_ test power output was sampled at 1 Hz. During the 30 s maximal sprint tests data was sampled at 0.5 Hz. Peak power and peak pedal cadence were defined as the highest power and cadence recorded by the cycle ergometer during each 30 s effort. Mean power was defined as the average of the power outputs during the 30 s all out efforts. Time to peak power was measured from the start of an effort to the time when the subject reached peak power.

### Statistical Analyses

Data modeling involved point estimation of peak and mean power response to stochastic and steady-state cycling protocols, and interval estimates of the uncertainty about the value of these parameters. A statistical approach using magnitude-based inferences and precision of estimation was used to determine practical/clinical significance of effects (Hopkins, 2017). Mean effects of the variable and constant power strategies and their 90% confidence limits (CL) were estimated via the unequal-variances *t*-statistic computed for change scores between pre- and post-tests of the two groups. Each subject’s change score was expressed as a percentage of baseline score via analysis of log-transformed values, in order to reduce bias arising from non-uniformity of error. The magnitude of difference between the two groups was expressed as a standardized effect size. The criteria to interpret the magnitude of effects were: <0.2 trivial, 0.2–0.6 small, 0.6–1.2 moderate, 1.2–2.0 large, and >2.0 very large (Hopkins, 2000).

For mean power output, we estimated the smallest worthwhile effect in this cohort of well-trained (but not elite) cyclists as 0.5 × 2 × 2.5 = 2.5% using the method outlined by Paton and Hopkins (2001) where 0.5 is the default smallest worthwhile proportion of the typical within-subject variability in time-based performance tasks or events (Hopkins et al., 1999), 2.0% is the estimated typical within-subject variability (% coefficient of variation) of well-trained road cyclists (Malcata and Hopkins, 2014), and 2.5 is the constant for conversion of performance time to power output (Paton and Hopkins, 2001; Malcata and Hopkins, 2014). When the 90% CL concurrently crossed the thresholds for the smallest meaningful decrement and improvement, the effect was deemed unclear. Standardized scores for correlation were interpreted according to a scale of magnitudes: <0.1 trivial, 0.1–0.3 small, 0.3–0.5 moderate, 0.5–0.7 large (Hopkins et al., 2009). A correlation was deemed unclear if the confidence interval spanned both -0.1 and +0.1 values. A sample size of 11 subjects was deemed appropriate in a single group cross-over design assuming a smallest worthwhile difference in mean power output of 2.5%, a typical error of 2.0%, and type I and II errors of 5 and 25% respectively. Descriptive data are reported as mean ± standard deviation (SD).

## Results

### Physiological and Performance Characteristics

The well-trained nature of the subject cohort was indicated by the values of maximal aerobic power and mean power output. Maximal aerobic power was 389 ± 32 W (mean ± SD) and peak B_La_ at the end of the test was 12.4 ± 2.3 mmol L^-1^ with a HR_peak_ of 189 ± 9 b min^-1^. Mean power output during the 1 h cycle was 233 ± 19 W for CON and 234 ± 20 W during VAR for all participants. The higher variability of the 1 h VAR protocol was indicated by a coefficient of variation (%CV) in power of 50% with an SD in power output of 117 ± 9 W. In contrast, the %CV during the 1 h CON protocol was only 9% with an SD in power output of 22 ± 5 W. Mean pedal rate was 94 ± 4 rev.min^-1^ and 95 ± 4 rev.min^-1^ for CON and VAR, respectively. There were trivial differences between CON and VAR in mean power and pedal rate.

### Variable Versus Constant Cycling

**FIGURE 2:**
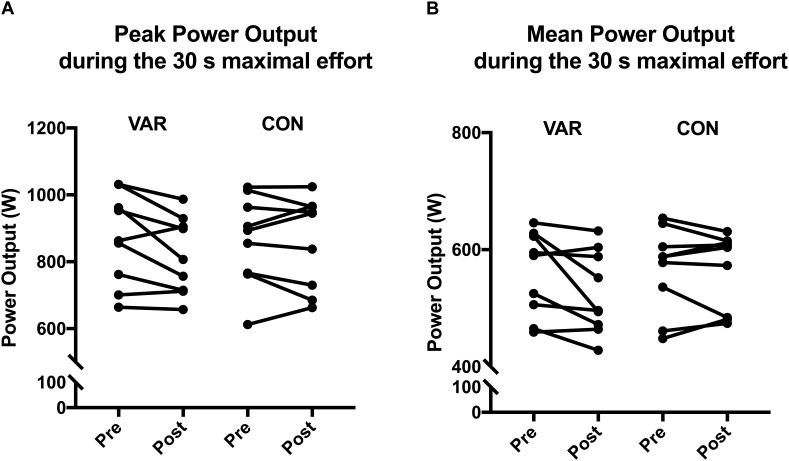
**(A)** The decrease in peak power output (watts) from the 30 s maximal effort before and after the 1 h constant (CON) and variable (VAR) power profiles. **(B)** The decrease in mean power output (watts) from the 30 s maximal effort before and after the 1 h constant (CON) and variable (VAR) power profiles.

The variability in power output during VAR hindered the ability to produce short-term maximal power output after 1 h of cycling compared to the constant power trial. Peak power during the 30 s sprint prior to the 1 h trials was similar: 866 ± 134 W (mean ± SD) for CON and 869 ± 137 W for VAR. There was a small difference in the change of peak power output between VAR and CON (-0.45 ± 0.37; standardized difference ± 90% CL) (Figure 2A). The 1 h VAR cycling decreased peak power output (-5.1 ± 6.1%; % difference ± 90%CL) and mean power output (-5.9 ± 5.2%) generated during the post-trial 30 s sprint (Table 1). Mean power output during the 30 s sprint prior to the 1 h cycle trials were also similar for CON (567 ± 73 W; mean ± SD) and VAR (560 ± 72 W). There was a small difference in the change of mean power output between VAR and CON (-0.33 ± 0.37; standardized difference ± 90%CL). After the VAR trial the mean power output was also lower by ∼6% to 526 ± 71 W (mean ± SD) with only a trivial change after CON to 565 ± 66 W (Figure 2B).

**Table 1:** Differences in peak power, mean power, peak pedal cadence and time to peak pedal cadence during the 30 s maximal sprint before and after 1 h constant and variable power cycling.

	% Change after 1 h trial ( ± 90% CL)		% Difference ( ± 90% CL)	Standardized difference ( ± 90% CL) qualitative inference
	Constant power	Variable power		
Peak power output (W)	-0.5 ± 6.4	-5.6 ± 7.3	-5.1 ± 6.1	-0.33 ± 0.37, small
Mean power output (W)	-0.3 ± 5.4	-6.1 ± 8.6	-5.9 ± 5.2	-0.45 ± 0.37, small
Peak pedal cadence (rpm)	-0.1 ± 10.7	-4.1 ± 10.8	-4.0 ± 8.7	-0.61 ± 1.25, unclear
Time to peak power (s)	-2.0 ± 38	1.0 ± 38	3.0 ± 17	0.08 ± 0.48, trivial


Time to peak power output and peak cadence before and after the 1 h cycle remained very similar for both CON (10.9 ± 2.0 vs. 10.9 ± 2.9 s and 126 ± 14 vs. 126 ± 6 rev.min^-1^) and VAR (10.4 ± 2.3 vs. 10.7 ± 3.4 s and 128 ± 12 vs. 123 ± 9 rev.min^-1^). A moderate relationship (*r* = -0.53, -0.83 to 0.03 90% confidence interval) between the relative maximal power output (W⋅kg^-1^) and the decrease in mean power output during the 30 s sprint was evident after the 1 h VAR condition. Similarly, there was a moderate relationship (*r* = -0.65, -0.83 to 0.05, 90% confidence interval) between triathletes/cyclists who had higher relative PPO (W⋅kg^-1^) during the 30 s sprint before the 1 h VAR having a larger decrease in PPO afterward.

## Discussion

Variable power cycling resembling criterium-style racing (bunch riding) and some track cycling events such as the scratch or points race, reduced end maximal sprinting capacity by ∼6%. This decrease in the ability to produce maximal power output during the latter stages of criterium-style races would be detrimental in the fight for a final sprint, often the way the race is decided, negatively impacting the final outcome of the race. The ∼6% decrease in 30 s peak and mean power output we observed at the end of the 1 h VAR protocol could translate to a reduced ability to fight for the top positions in a world championship or Olympics Games when races can be decided in the last 20–25 s of the race. Both road and track cycling events such as the scratch and points race contain multiple high intensity efforts during the event, given the interplay of technical and tactical factors.

The investigation of variable and constant power cycling required development of a suitable laboratory-based cycling protocol with acceptable content and face validity. The relative mean power output for the 1 h cycle trial in this study (∼60% maximal aerobic power) is comparable to that described in criterium style races by Ebert et al. (2006). The frequent high intensity efforts featured in our 1 h VAR protocol by the numerous supra-maximal efforts (>maximal aerobic power) simulated the power fluctuations and duration of effort observed in real-world cycling performances. This study overcomes some of the shortcomings of previous studies implementing short 30 min protocols (Lepers et al., 2008; Theurel and Lepers, 2008) or had not included multiple short (10–30 s) high intensity efforts (Suriano et al., 2010) common in criterium races. Furthermore, the power distribution implemented during the variable power protocol in the present study is similar to the protocol resembling the cycle section of draft legal triathlon races (Etxebarria et al., 2013). Consequently, the decrement in peak and mean power output during a maximal sprint in the present study is representative in terms of content and face validity, and applicable to these sporting situations. More specifically, slower swimmers who do not make the leading pack (during subsequent cycling) and tend to increase their power output during the latter stages of the cycling section to breach the gap (Vleck et al., 2008), might be negatively affected by the higher cycling power output and compromize their chance to ‘get into’ the race or keep being a contender for the top positions.

The greater decrease in peak power output in the 30 s maximal sprint after VAR presumably reflects accumulated fatigue from the repeated high intensity peaks observed in races. Fatigue caused by repetitive intermittent and high intensity exercise is influenced by a combination of metabolic (Westgarth-Taylor et al., 1997; Stepto et al., 2001; Allen et al., 2008) and neuromuscular factors (Gandevia, 2001). Variable power cycling has also different muscle recruitment patterns (Palmer et al., 1999; Suriano et al., 2010) and metabolic responses (Palmer et al., 1999) than cycling at constant power. Variable power cycling that includes supra maximal intensities decrease maximal voluntary contraction torque and activation (Billaut et al., 2006; Theurel and Lepers, 2008) but not when lower (60 to 90% maximal aerobic power) exercise intensities are employed (Lepers et al., 2008). The evidence presented in this study should inform coaches and athletes of the areas to focus their training on when competing under criterium style race demands, repeat sprint-ability, and power variability.

Variable and constant power cycling appear to yield a similar decrease in total glycogen (Brickley et al., 2007). However, cycling at variable power elicits a greater level of glycogen depletion in type II muscle fibers (Palmer et al., 1999; Suriano et al., 2010), the same pool of muscle fibers that would have been targeted during a 30 s maximal sprint. Athletes with a higher PPO are likely to have a higher percentage of these fast-twitch fibers. Consequently these athletes are more likely to fatigue after several sprints, inducing a greater drop-off in PPO after VAR, which an outcome we observed in this study. Therefore, constant power cycling is likely to spare higher levels of PCr (non-oxidative path) for the post-trial 30 s effort as high intensity efforts rely on non-oxidative ATP resynthesis pathways (Gastin and Lawson, 1994; Bogdanis et al., 1996). The high intensity efforts involved in VAR induce three times the blood lactate concentration at the end of the 1 h protocol compared with CON (Etxebarria et al., 2013). The selective depletion of glycogen during variable power cycling and reported higher glucose oxidation (Palmer et al., 1999) could be the explanation why fluctuating power output is detrimental to end race performance and/or the early stages of the subsequent running section in triathlon (Etxebarria et al., 2014a). Future studies should investigate relationships between patterns of glycogen depletion, power profiles, and potentially dietary manipulation as a strategy for improving race performance.

Given the wide range of cycling intensities coupled with frequent changes in pace (similar to those experienced in criterium and triathlon), the deleterious effects of variable power cycling on short-term maximal power generation capacities, are applicable to real-world race situation. The ∼6% reduction in the ability to generate power after variable power cycling could have negative implications for the late stages of a criterium style cycle race. A geographically (hills) and technically (multiple corners) challenging cycling course in which major competitions are race under could translate to early fatigue and loss of medal hope. The cycling course for the Tokyo 2020 Olympics and Paralympics will start in the metropolitan area and already described as ‘startingly testing course’ for the road cycling and time trial events^1^. Similarly, the triathlon course for the cycling section is based on a 5 km closed circuit course with several 360 and 180 degree turns in each lap^2^. Therefore, athletes could benefit from using a similar interval training protocol to the VAR intervention in to gain specific adaptations to race demands during the Tokyo games.

Further research is needed to investigate the mechanisms explaining this greater reduction in peak and mean power output during a 30 s all-out effort after a relatively short (∼60–90 min), variable power cycle bout. However, there are several specific training strategies that could help in promoting specific adaptations to technical courses inducing variable power output such as high intensity interval training (Etxebarria et al., 2014a) and improving cycling technical competency (Babault et al., 2018). These strategies are especially important for triathletes, who do not spend as much time on the bike to develop bunch-riding and technical skills as specialist cyclists do.

### Practical Applications

A diminished ability to generate peak power outputs could lead to a variety of detrimental race situations for athletes, including missing an attacking opportunity (defensive shortcoming) or failing to create a breakaway to establish a leading gap (attacking shortcoming) over an opponent or group of opponents. Coaches and athletes should consider the 1 h sport-specific cycling protocol as a useful training option for cyclists to prepare for races. This type of training should increase the ability to produce repeat sprints with small reductions in peak power toward the end of road races, as well as track cycling events such as the scratch or the points race. The more powerful cyclists were most vulnerable to losing their sprint-ability after fluctuating power cycling in this study – these athletes could benefit from further developing their aerobic capacity.

The cycling demands during major competitions such as the Olympic Games and World Championships are often dictated by other competitors’ tactics and performances, and/or the technical nature of the course that induce high intensity efforts and multiple changes in cycling pace. Improving cycling skills in preparation for highly technical courses may be advantageous to limit abrupt decelerations and consequent sharp accelerations out of corners. These skills would enable athletes to sustain a higher velocity for less power produced, increased control for peloton management and ‘saving the legs’ for a final sprint. The aim of athletes competing in criterium style cycling courses should be to combine increased sprint-ability, minimizing power variability, promoting recovery between sprints, and optimizing technical skills.

## Conclusion

Cycling at race-specific variable power output for 1 h appears to decrease the ability to generate short-term (30 s) maximal power output compared to cycling at constant power for the duration. Athletes, coaches, and staff should evaluate training and race-day strategies to better maintain the final sprint or end spurt.

## Ethics Statement

The study was approved by the ethics committee for human research at Loughborough University.

## Author Contributions

NE, SI, and RF devised the study design. NE conducted the data collection and processing. All authors participated in interpretation of the data, preparation of the written manuscript, and read and approved the final manuscript.

## Conflict of Interest Statement

The authors declare that the research was conducted in the absence of any commercial or financial relationships that could be construed as a potential conflict of interest.

## Footnotes

1 https://www.cyclingweekly.com/news/racing/olympics/olympic-cycling-race-route-357988

2 https://tokyo2020.org/en/games/sport/olympic/triathlon/
